# Characterization of the complete chloroplast genome of *Lindera aggregate*

**DOI:** 10.1080/23802359.2021.1978890

**Published:** 2021-09-24

**Authors:** Lixia Yuan, Yan Sun, Shuanglin Zhou, Furong Wang, Chonglu Sun

**Affiliations:** Zhejiang Pharmaceutical College, Ningbo, Zhejiang, China

**Keywords:** *Lindera aggregate*, plastid genome structure phylogeny

## Abstract

In this study, we sequenced the complete chloroplast genome of *Lindera aggregate* (Sims) Kosterm., an important Chinese herbal medicine. The complete chloroplast genome with a size of 152,714 bp in length, contained two inverted repeats (IRa and IRb) regions of 20,090 bp each, which were separated by a large single copy (LSC, 93,743 bp) regions and a small single copy (SSC, 18,791 bp) regions, the overall GC content was 42.84%. The chloroplast genome contained 122 genes, 77 protein-coding, 37 tRNA, and 8 rRNA genes. The phylogenetic tree showed that *Lindera aggregate* (Sims) Kosterm. has a close relationship with *Lindera chuni*.

*Lindera aggregate* (Sims) Kosterm. is a traditional Chinese medicine, belong to *Lindera* (Lauraceae), distribute in the southern area of the Yangtze River in China, Japan, and other southeastern Asian countries (Chen et al. 2012). The root of this plant has an anti-inflammatory analgesic effect, is food for new national resources, non-toxic and long-term use (Tao and Jiang 2008). The recent finding indicated that Lindera aggregate (Sims) Kosterm. has potential therapeutic value in the treatment of hyperlipidemia s (Wang et al. 2020). In the present study, we characterize the complete chloroplast genome of *Lindera aggregate* (Sims) Kosterm. and provide basic data for studying the phylogenetic relationships in Lauraceae.

The mature leaves of *Lindera aggregate* (Sims) Kosterm. from Hangzhou county (119°30′ E, 29°55′ N), Zhejiang Province, China. Total genomic DNA was extracted by Plant Genomic DNA Kit. The sample was deposited at the Zhejiang Pharmaceutical College Ningbo, China (Tianlin Lou . e-mail: tianling1126@sina. com) under Voucher No. HZWY-202006, and DNA compounds were stored at −20 °C. Sequenced through Illumina genome analyzer (Hiseq PE150), the genomic DNA was isolated and extracted to an average 300 bp paired-end(PE) library with the Illumina Hiseq platform. In all, 14,103,993 of PE150-bp raw reads were obtained, chloroplast genome-related reads were sieved by mapping to the closer species *Lindera chuni*. Contigs, assembled using SOAPdenovo (Luo et al. 2012), were sorted and joined into a single-draft sequence using Geneious (Kearse et al. 2012), by comparison with the chloroplast sequence of *Lindera chuni* as a reference. The resultant clean reads were then employed to assemble the chloroplast genome using the program NOVOPlasty (Dierckxsens et al. 2017) with *L. chuni* (GenBank: NC045254.1) as the reference. The genes in the chloroplast genome were predicted by using GeSeq (Tillich et al. 2017) and corrected by Blast search.

The chloroplast genome of *Lindera aggregata* (Sims) Kosterm. was a circular molecular genome with a size of 152,714 bp in length, of which the overall GC content was 42.84%. It contained two inverted repeats, IRa and IRb, regions of 20,090 bp, which were separated by a large single copy (LSC) region of 93,743 bp and a small single copy (SSC) region of 18,791 bp. The chloroplast genome contained 122 genes, 77 protein-coding, 37 tRNA, and 8 rRNA genes.

To resolve the phylogenetic relationships of *Lindera aggregate* (Sims) Kosterm. with other species in Lauraceae, we download complete chloroplast DNA sequences of 15 related species in Lauraceae as ingroups and two species from Calycanthaceae as outgroups. These sequences were trimmed properly by trimAl (Capella-Gutierrez et al. 2009) and aligned by MAFFT (Katoh et al. 2002). The phylogenetic tree was constructed with MEGA7.0 (Kumar et al. 2016). Bootstrap values were calculated from 1000 replicate analysis. The results showed that *Lindera aggregate* (Sims) Kosterm. as a paraphyletic group in chloroplast phylogenomics, and it had a close relationship with *Lindera chuni* (NC045254) in the phylogenetic tree (Figure 1).

**Figure 1.**
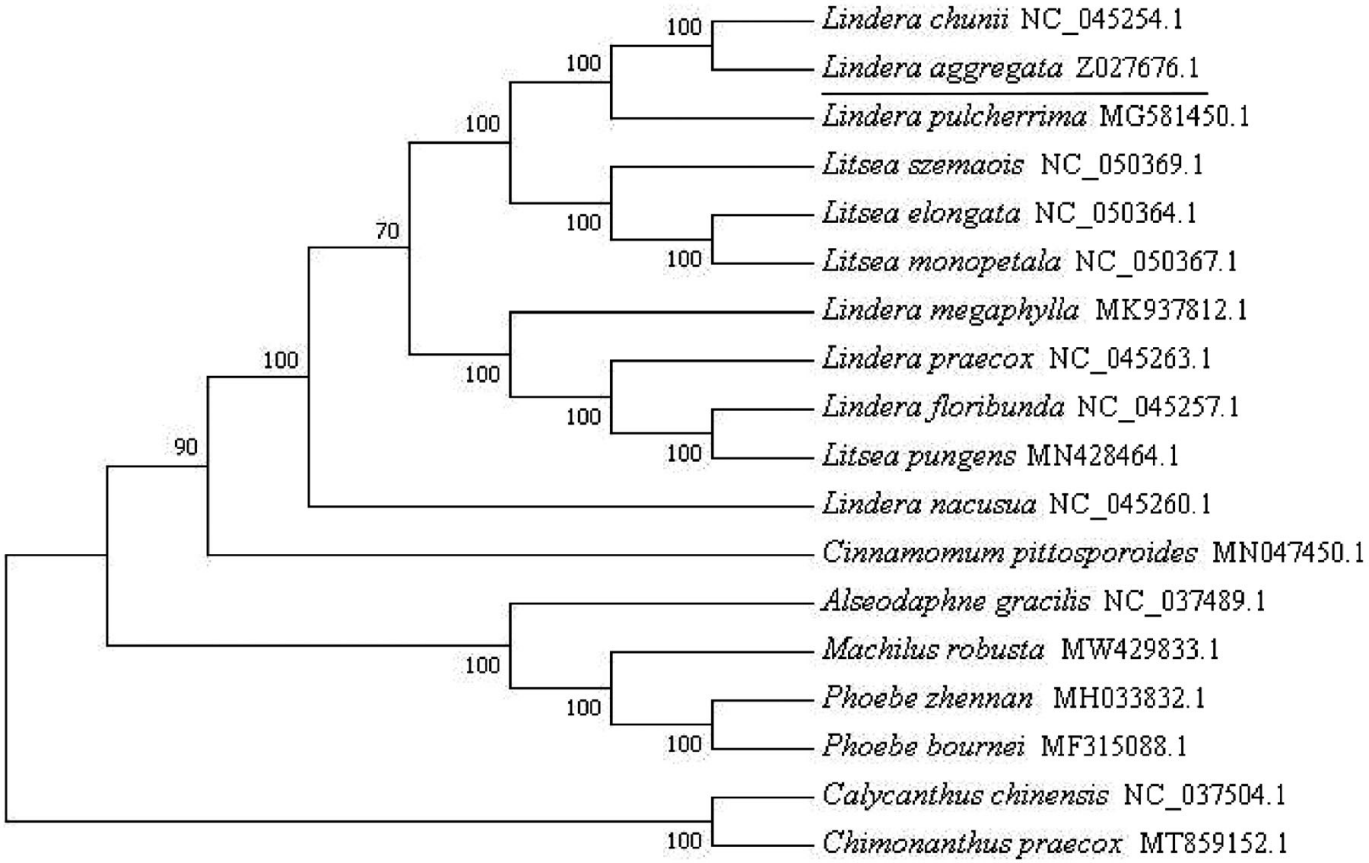
Phylogenetic tree inferred from16 complete Lauraceae genomes. Two Calycanthaceae species were used as outgroups.

## Data Availability

The genome sequence data that support the findings of this study are openly available in GenBank of NCBI at https://www.ncbi.nlm.nih.gov/ under the accession no. MZ027676.1. The associated BioProject, SRA, and Bio-Sample numbers are PRJNA728200, SRR14532211, and SAMN19071605, respectively.
